# Supplementary material for: Respiratory Tract Cancer Incidences across Industry Groups: A Nationwide Cohort Study with More Than 70 Million Person-Years of Follow-Up
Source: Cancers (Basel). 2022 Oct 25;14(21):5219. doi: 10.3390/cancers14215219 (PMC9658191; doi:10.3390/cancers14215219)
Supplement: Supplementary file 1 [file cancers-14-05219-s001.zip › cancers-1947815-supplementary.pdf]

Table S1. Standardized incidence ratio (SIR) and confidence intervals (CIs) of laryngeal cancer for each industry group

KSIC code	Industry group	Male	
		Observed cases / person-year	SIR [95% CI]
107	Manufacture of other food products	6 / 347791	1.16 [0.43-2.52]
139	Manufacture of other made-up textile articles, except apparel	6 / 111657	2.45 [0.90-5.33]
222	Manufacture of plastics products	8 / 219516	1.86 [0.80-3.67]
241	Manufacture of basic iron and steel	7 / 488469	0.66 [0.26-1.36]
251	Manufacture of structural metal products, tanks, reservoirs and steam generators	6 / 293835	1.10 [0.40-2.39]
259	Manufacture of other fabricated metal products; metalworking service activities	9 / 426352	1.09 [0.50-2.06]
292	Manufacture of special-purpose machinery	14 / 535930	1.64 [0.90-2.76]
301	Manufacture of motor vehicles and engines for motor vehicles	9 / 825983	0.76 [0.35-1.45]
303	Manufacture of parts and accessories for motor vehicles (new products)	12 / 840595	0.99 [0.51-1.73]
311	Building of ships and boats	12 / 693555	0.83 [0.43-1.44]
339	Other manufacturing n.e.c.	95 / 5138809	1.09 [0.88-1.33]
351	Electric power generation, transmission and distribution	12 / 462108	1.13 [0.59-1.98]
411	Building construction	38 / 1618515	1.03 [0.73-1.41]
412	Heavy and civil engineering construction	23 / 966604	0.96 [0.61-1.43]
421	Site preparation and construction of foundation works and structures	16 / 511087	1.27 [0.73-2.07]
423	Electrical and communication works	8 / 428606	0.89 [0.38-1.75]
424	Building completion and finishing	10 / 432093	1.04 [0.50-1.91]
461	Wholesale on a fee or contract basis	14 / 511197	1.58 [0.86-2.65]
464	Wholesale of household goods	8 / 291291	1.54 [0.66-3.03]
467	Other specialized wholesale	20 / 725680	1.44 [0.88-2.23]
471	Retail sale in non-specialized stores	18 / 1159171	0.94 [0.56-1.48]
477	Retail sale of fuels	6 / 129141	1.46 [0.54-3.18]
478	Retail sale in other goods in specialized stores	12 / 594374	1.21 [0.63-2.11]
492	Passenger land transport,	21 / 308510	1.65 [1.02-2.53]

	except transport via railways		
501	Sea and coastal water transport	8 / 158776	1.27 [0.55-2.49]
529	Support activities for transportation	95 / 2184768	1.16 [0.94-1.42]
561	Restaurants and mobile food service activities	7 / 400235	0.99 [0.40-2.04]
612	Telecommunications	7 / 385146	1.06 [0.42-2.18]
641	Banking and savings institutions	17 / 1355152	0.74 [0.43-1.18]
681	Real estate activities with own or leased property	43 / 1261326	0.96 [0.69-1.29]
682	Real estate activities on a fee or contract basis	37 / 564051	1.10 [0.77-1.52]
721	Architectural, engineering and related technical services	11 / 600972	0.77 [0.38-1.37]
741	Combined facilities support activities	9 / 191038	1.06 [0.49-2.02]
742	Cleaning and pest control services of building and industrial facilities	12 / 170184	1.15 [0.60-2.01]
759	Other business support services	23 / 1037580	0.79 [0.50-1.19]
841	Executive, legislative and general government support	10 / 239610	1.24 [0.59-2.28]
872	Non-residential welfare facilities	12 / 297117	1.79 [0.92-3.12]
912	Amusement parks and other recreation activities	8 / 351569	0.67 [0.29-1.31]
949	Other membership organizations	24 / 779792	0.99 [0.64-1.48]
969	Other personal service activities n.e.c.	25 / 685091	1.32 [0.85-1.94]
982	Undifferentiated service-producing activities of private households for own use	15 / 150551	1.53 [0.86-2.52]

Table S2. Standardized incidence ratio (SIR) and confidence intervals (CIs) of lung cancer for each industry group

KSIC code	Industry group	Male		Female	
		Observed cases / person-year	SIR [95% CI]	Observed cases / person-year	SIR [95% CI]
012	Animal production	19 / 36266	1.72 [1.03-2.68]	-	-
014	Services incidental to growing of crops and raising of animals	8 / 30285	1.00 [0.43-1.96]	-	-
020	Forestry	11 / 17780	1.48 [0.74-2.65]	-	-
031	Fishing	21 / 33588	1.70 [1.05-2.60]	-	-
032	Aquaculture and services incidental to fishing and aquaculture	7 / 15591	1.66 [0.67-3.41]	-	-
051	Mining of coal and lignite	43 / 68719	1.69 [1.22-2.27]	-	-
071	Quarrying of stone, sand and clay	12 / 22453	1.32 [0.68-2.30]	-	-
101	Slaughtering of livestock, processing and preserving of meat and meat products	16 / 52684	1.21 [0.69-1.97]	6 / 44963	0.65 [0.24-1.41]
102	Processing and preserving of fish, crustaceans, molluscs and seaweeds	7 / 19335	1.23 [0.49-2.53]	8 / 27282	1.34 [0.58-2.63]
103	Processing and preserving of fruit and vegetables	7 / 15116	1.72 [0.69-3.54]	-	-
104	Manufacture of vegetable and animal oils and fats	7 / 28081	1.28 [0.51-2.63]	-	-
107	Manufacture of other food products	64 / 347713	1.10 [0.85-1.41]	27 / 247726	0.76 [0.50-1.10]
108	Manufacture of prepared animal feeds and feed additives	6 / 26184	0.80 [0.29-1.74]	-	-
112	Manufacture of ice and non-alcoholic beverages; production of mineral waters	6 / 25249	1.65 [0.60-3.59]	-	-
131	Spinning of textiles and processing of threads and yarns	25 / 65416	1.26 [0.82-1.87]	10 / 44174	1.45 [0.69-2.66]
132	Weaving of textiles and manufacture of textile products	41 / 192989	0.89 [0.64-1.21]	7 / 110396	0.49 [0.20-1.00]
133	Manufacture of knitted and crocheted fabrics	10 / 37601	1.03 [0.49-1.90]	-	-
134	Dyeing and finishing of textiles and wearing apparel	26 / 68227	1.36 [0.89-1.99]	-	-
139	Manufacture of other made-up textile articles, except apparel	29 / 111623	1.08 [0.72-1.55]	12 / 83042	1.05 [0.54-1.83]
141	Manufacture of sewn wearing apparel, except fur apparel	19 / 95065	0.96 [0.58-1.50]	14 / 116537	0.94 [0.51-1.57]
144	Manufacture of apparel	7 / 14751	2.31 [0.93-4.76]	-	-

	accessories				
152	Manufacture of footwear and parts of footwear	15 / 49966	1.09 [0.61-1.80]	-	-
161	Sawmilling and planing of wood	34 / 75615	1.37 [0.95-1.91]	-	-
162	Manufacture of wood products	24 / 77058	1.16 [0.75-1.73]	-	-
171	Manufacture of pulp, paper and paperboard	36 / 136638	1.07 [0.75-1.48]	-	-
172	Manufacture of corrugated paper, paper boxes and paper containers	18 / 52997	1.32 [0.78-2.08]	-	-
181	Printing and service activities related to printing	81 / 431308	0.94 [0.75-1.17]	10 / 248244	0.51 [0.24-0.93]
192	Manufacture of refined petroleum products	15 / 63699	1.30 [0.72-2.14]	-	-
201	Manufacture of basic chemicals	61 / 320428	1.07 [0.82-1.38]	6 / 56526	1.38 [0.51-3.01]
202	Manufacture of plastics and synthetic rubber in primary forms	49 / 248962	0.97 [0.72-1.28]	6 / 74625	0.55 [0.20-1.20]
203	Manufacture of fertilizers, pesticides, germicides and insecticides	6 / 12857	1.61 [0.59-3.50]	-	-
204	Manufacture of other chemical products	39 / 225843	1.04 [0.74-1.42]	6 / 78467	0.87 [0.32-1.90]
205	Manufacture of man-made fibers	7 / 30169	1.16 [0.46-2.38]	-	-
222	Manufacture of plastics products	53 / 219427	1.12 [0.84-1.47]	-	-
231	Manufacture of glass and glass products	14 / 66204	1.11 [0.61-1.86]	-	-
233	Manufacture of cement, lime, plaster and its products	25 / 83947	1.14 [0.74-1.68]	-	-
239	Manufacture of other non-metallic mineral products	33 / 134409	0.92 [0.63-1.29]	-	-
241	Manufacture of basic iron and steel	123 / 488273	1.06 [0.88-1.27]	9 / 59960	1.28 [0.59-2.44]
242	Manufacture of basic precious and non-ferrous metals	23 / 110404	0.83 [0.53-1.25]	-	-
243	Casting of metals	35 / 151226	0.96 [0.67-1.33]	-	-
251	Manufacture of structural metal products, tanks, reservoirs and steam generators	66 / 293710	1.10 [0.85-1.40]	9 / 56096	1.33 [0.61-2.53]
259	Manufacture of other fabricated metal products; metalworking service activities	100 / 426163	1.10 [0.90-1.34]	10 / 95398	0.78 [0.37-1.43]
262	Manufacture of electronic components	44 / 369238	0.93 [0.67-1.24]	25 / 224910	1.03 [0.67-1.52]
263	Manufacture of computers and peripheral equipment	7 / 59051	0.96 [0.38-1.97]	-	-
264	Manufacture of communication and broadcasting apparatuses	18 / 152791	1.03 [0.61-1.63]	-	-

265	Manufacture of electronic video and audio equipment	63 / 672861	0.99 [0.76-1.27]	15 / 237762	0.89 [0.50-1.46]
271	Manufacture of medical and dental instruments and supplies	7 / 45959	1.07 [0.43-2.21]	-	-
281	Manufacture of electric motors, generators, transformers and electricity distribution and control apparatus	45 / 271180	0.94 [0.69-1.26]	-	-
283	Manufacture of insulated wires and cables	9 / 81992	0.60 [0.27-1.14]	-	-
284	Manufacture of electric tubes and bulbs and lighting equipment	9 / 68686	0.76 [0.35-1.44]	-	-
285	Manufacture of domestic appliances	8 / 39853	0.88 [0.38-1.73]	-	-
291	Manufacture of general purpose machinery	100 / 527105	0.90 [0.73-1.09]	7 / 93981	0.64 [0.26-1.31]
292	Manufacture of special-purpose machinery	107 / 535747	1.13 [0.92-1.36]	18 / 119876	1.34 [0.79-2.12]
301	Manufacture of motor vehicles and engines for motor vehicles	112 / 825743	0.83 [0.69-1.00]	-	-
303	Manufacture of parts and accessories for motor vehicles (new products)	118 / 840368	0.87 [0.72-1.04]	28 / 255872	0.74 [0.49-1.06]
311	Building of ships and boats	149 / 693297	0.96 [0.81-1.12]	13 / 66119	1.65 [0.88-2.83]
312	Manufacture of railway locomotives and rolling stock	9 / 38878	0.88 [0.40-1.67]	-	-
319	Manufacture of other transport equipment	14 / 73456	0.89 [0.48-1.49]	-	-
320	Manufacture of furniture	31 / 127946	1.07 [0.73-1.52]	-	-
339	Other manufacturing n.e.c.	998 / 5137041	1.03 [0.97-1.10]	170 / 1614762	0.93 [0.79-1.08]
351	Electric power generation, transmission and distribution	112 / 461853	0.99 [0.81-1.19]	-	-
352	Manufacture of gas; distribution of gaseous fuel through mains	12 / 40067	1.29 [0.67-2.25]	-	-
360	Water supply	9 / 32214	1.15 [0.52-2.18]	-	-
381	Waste collection	19 / 29823	1.49 [0.90-2.33]	-	-
382	Waste treatment and disposal services	7 / 26003	0.73 [0.29-1.50]	-	-
383	Dismantling and sorting recoverable materials; materials recovery	13 / 48682	0.89 [0.48-1.53]	-	-
411	Building construction	412 / 1617825	1.02 [0.92-1.12]	27 / 301764	0.90 [0.59-1.31]
412	Heavy and civil engineering construction	308 / 966047	1.17 [1.05-1.31]	17 / 179191	0.94 [0.55-1.51]
421	Site preparation and construction of foundation works and structures	143 / 510867	1.04 [0.88-1.23]	12 / 99471	1.19 [0.62-2.09]
422	Construction of installing building equipment	28 / 108927	0.97 [0.65-1.41]	-	-

423	Electrical and communication works	110 / 428391	1.11 [0.91-1.34]	-	-
424	Building completion and finishing	107 / 431903	1.01 [0.83-1.22]	8 / 122759	0.66 [0.28-1.30]
426	Renting of construction machinery and equipment with operator	29 / 85185	1.21 [0.81-1.74]	-	-
452	Sale of motor vehicle parts and accessories	35 / 182717	1.10 [0.77-1.53]	-	-
461	Wholesale on a fee or contract basis	109 / 510998	1.10 [0.91-1.33]	34 / 316644	1.22 [0.84-1.70]
462	Wholesale of agricultural raw materials and live animals and plants	11 / 58578	0.92 [0.46-1.65]	-	-
463	Wholesale of food, beverages and tobaccos	49 / 224121	1.09 [0.81-1.44]	16 / 121200	0.92 [0.52-1.49]
464	Wholesale of household goods	49 / 291211	0.85 [0.63-1.12]	23 / 199638	1.18 [0.75-1.77]
465	Wholesale of machinery, equipment and supplies	77 / 393946	0.91 [0.71-1.13]	22 / 118943	1.88 [1.18-2.85]
466	Wholesale of construction materials, hardware and heating and air conditioning equipment	47 / 144916	1.26 [0.92-1.67]	-	-
467	Other specialized wholesale	147 / 725506	0.96 [0.81-1.13]	37 / 318360	1.26 [0.89-1.73]
468	Non-specialized wholesale trade	16 / 52852	1.47 [0.84-2.38]	-	-
471	Retail sale in non-specialized stores	180 / 1158901	0.84 [0.72-0.97]	65 / 668639	1.02 [0.79-1.30]
472	Retail sale of foods, beverages and tobacco in specialized stores	19 / 71658	1.21 [0.73-1.90]	8 / 45167	1.24 [0.54-2.45]
474	Retail sale of textiles, clothing, footwear and leather goods	22 / 108333	1.04 [0.65-1.57]	16 / 111346	1.55 [0.89-2.52]
475	Retail sale of other household equipment	34 / 211776	0.88 [0.61-1.23]	9 / 70480	1.32 [0.60-2.51]
476	Retail sale of cultural, amusement and recreation goods	8 / 61273	0.76 [0.33-1.51]	-	-
477	Retail sale of fuels	36 / 129088	0.81 [0.57-1.12]	10 / 36692	1.75 [0.84-3.22]
478	Retail sale in other goods in specialized stores	102 / 594205	0.92 [0.75-1.12]	31 / 378130	0.82 [0.56-1.17]
479	Retail sale not in stores	13 / 89769	1.10 [0.59-1.89]	6 / 54442	1.68 [0.62-3.67]
491	Transport via railways	43 / 205611	0.88 [0.64-1.18]	-	-
492	Passenger land transport, except transport via railways	151 / 308279	1.12 [0.95-1.31]	8 / 43397	1.76 [0.76-3.47]
493	Freight transport by road	40 / 119860	1.35 [0.96-1.83]	-	-
501	Sea and coastal water transport	60 / 158706	0.89 [0.68-1.14]	-	-
529	Support activities for transportation	1061 / 2183034	1.22 [1.15-1.30]	19 / 311331	0.69 [0.42-1.08]
551	General accommodation and accommodation with cooking facilities	36 / 204577	0.92 [0.64-1.27]	21 / 154559	0.92 [0.57-1.40]

561	Restaurants and mobile food service activities	75 / 400093	0.96 [0.76-1.21]	125 / 634621	1.06 [0.88-1.26]
562	Drinking places and non-alcoholic beverages places	6 / 24252	1.28 [0.47-2.78]	-	-
591	Motion picture, video and broadcast program activities	6 / 51806	0.83 [0.30-1.80]	-	-
612	Telecommunications	51 / 385041	0.70 [0.52-0.92]	-	-
620	Computer programming, consultancy and related activities	31 / 446407	0.83 [0.56-1.17]	7 / 161688	1.11 [0.44-2.28]
631	Data processing, hosting and related activities; web portals	25 / 453865	0.69 [0.45-1.02]	7 / 154680	1.00 [0.40-2.07]
639	Other information service activities	22 / 180145	1.09 [0.69-1.66]	-	-
641	Banking and savings institutions	231 / 1354601	0.91 [0.79-1.03]	57 / 912772	1.28 [0.97-1.66]
651	Insurance	35 / 170144	1.18 [0.82-1.65]	7 / 112675	1.31 [0.53-2.71]
661	Activities auxiliary to financial service activities	13 / 72490	1.24 [0.66-2.12]	-	-
681	Real estate activities with own or leased property	475 / 1260525	0.97 [0.88-1.06]	94 / 548105	1.04 [0.84-1.27]
682	Real estate activities on a fee or contract basis	407 / 563482	1.11 [1.01-1.23]	59 / 272215	0.96 [0.73-1.24]
711	Legal services	59 / 225077	0.85 [0.65-1.10]	10 / 137887	1.35 [0.65-2.48]
712	Accounting, bookkeeping and auditing activities; tax consultancy	42 / 179002	0.88 [0.63-1.19]	13 / 234013	0.93 [0.49-1.58]
713	Advertising	13 / 119354	0.82 [0.44-1.40]	6 / 92532	1.16 [0.42-2.51]
715	Activities of head offices, management consultancy activities	9 / 80372	0.57 [0.26-1.08]	-	-
721	Architectural, engineering and related technical services	129 / 600729	0.82 [0.68-0.97]	14 / 148778	1.33 [0.73-2.23]
729	Other scientific and technical services	9 / 60198	0.75 [0.34-1.42]	-	-
739	Other professional, scientific and technical services n.e.c.	9 / 58272	0.84 [0.39-1.60]	-	-
741	Combined facilities support activities	108 / 190859	1.17 [0.96-1.42]	22 / 86571	1.01 [0.63-1.52]
742	Cleaning and pest control services of building and industrial facilities	139 / 169980	1.24 [1.04-1.46]	54 / 179741	1.02 [0.76-1.33]
751	Activities of employment placement agencies and provision of human resources	95 / 254652	1.02 [0.83-1.25]	31 / 271904	0.78 [0.53-1.10]
752	Activities of travel agencies and tour operators and tourist assistance activities	39 / 75153	1.41 [1.00-1.93]	-	-
753	Security, guard and detective services	80 / 111193	1.16 [0.92-1.45]	6 / 26050	0.79 [0.29-1.72]
759	Other business support services	329 / 1037035	1.04 [0.93-1.15]	106 / 809725	1.02 [0.84-1.24]

763	Renting of industrial machinery and equipment	8 / 88181	0.56 [0.24-1.10]	-	-
841	Executive, legislative and general government support	104 / 239418	1.21 [0.98-1.46]	6 / 132735	0.40 [0.15-0.88]
842	Administration of industrial and social policy of community	7 / 18729	1.13 [0.45-2.33]	-	-
851	Pre-primary and primary education	28 / 68627	1.02 [0.68-1.47]	89 / 1108083	0.87 [0.70-1.07]
852	Secondary education	13 / 50285	1.12 [0.60-1.92]	19 / 200365	0.94 [0.56-1.46]
853	Higher education	13 / 43601	1.13 [0.60-1.92]	13 / 90708	1.31 [0.70-2.24]
855	General educational institutes	14 / 120392	0.58 [0.32-0.97]	8 / 161666	0.69 [0.30-1.36]
856	Other educational institutes	46 / 172078	0.93 [0.68-1.25]	36 / 361095	1.08 [0.75-1.49]
861	Hospital activities	51 / 264366	1.03 [0.76-1.35]	70 / 637586	1.29 [1.01-1.63]
862	Medical and dental practice activities	109 / 483221	0.88 [0.72-1.06]	85 / 1152277	1.05 [0.84-1.30]
863	Public health centers	30 / 62411	0.88 [0.59-1.26]	7 / 47102	0.68 [0.27-1.39]
869	Other human health activities	6 / 29873	1.15 [0.42-2.51]	11 / 41399	2.08 [1.04-3.72]
871	Residential welfare facilities	15 / 61280	0.97 [0.54-1.60]	36 / 237772	0.84 [0.59-1.16]
872	Non-residential welfare facilities	62 / 297024	0.84 [0.65-1.08]	109 / 864306	1.13 [0.93-1.36]
901	Creative and arts related services	6 / 31001	1.61 [0.59-3.51]	-	-
902	Library, historical sites and buildings and similar recreation related services	9 / 17588	1.89 [0.86-3.58]	-	-
911	Sports services	27 / 92422	1.26 [0.83-1.83]	-	-
912	Amusement parks and other recreation activities	130 / 351331	0.99 [0.83-1.18]	28 / 222344	0.83 [0.55-1.20]
941	Business, employers and professional membership organizations	9 / 43967	0.84 [0.38-1.59]	-	-
949	Other membership organizations	251 / 779370	0.96 [0.84-1.08]	65 / 500843	0.93 [0.72-1.18]
951	Maintenance and repair services of computers and communication equipment	17 / 118322	0.86 [0.50-1.38]	-	-
952	Maintenance and repair services of motor vehicles and motorcycles	41 / 243794	1.02 [0.73-1.38]	-	-
961	Personal care services	8 / 28300	0.77 [0.33-1.52]	9 / 53327	1.31 [0.60-2.49]
969	Other personal service activities n.e.c.	207 / 684813	1.00 [0.87-1.14]	59 / 476140	0.92 [0.70-1.19]
982	Undifferentiated service-producing activities of private households for own use	114 / 150423	1.07 [0.89-1.29]	14 / 93891	0.86 [0.47-1.44]
990	Activities of extraterritorial organizations and bodies	23 / 70606	0.74 [0.47-1.11]	-	-
